# Demand for pneumococcal vaccination under subsidy program for the elderly in Japan

**DOI:** 10.1186/1472-6963-12-313

**Published:** 2012-09-12

**Authors:** Masahide Kondo, Mariko Yamamura, Shu-Ling Hoshi, Ichiro Okubo

**Affiliations:** 1 University of Tsukuba, Graduate School of Comprehensive Human Sciences, Doctoral Program in Human Care Science, Department of Health Care Policy and Management, 1-1-1 Tennoudai, Tsukuba, Ibaraki, Japan; 2 Hiroshima University, Graduate School of Education, Department of Mathematics Education, 1-1-1 Kagamiyama, Higashi-Hiroshima, Japan

**Keywords:** Demand, Elderly, Vaccination program, Pneumococcal polysaccharide vaccine (PPV), Price elasticity, Public relations, Subsidy

## Abstract

**Background:**

Vaccination programs often organize subsidies and public relations in order to obtain high uptake rates and coverage. However, effects of subsidies and public relations have not been studied well in the literature. In this study, the demand function of pneumococcal vaccination among the elderly in Japan is estimated, incorporating effects of public relations and subsidy.

**Methods:**

Using a data from a questionnaire survey sent to municipalities, the varying and constant elasticity models were applied to estimate the demand function. The response variable is the uptake rate. Explanatory variables are: subsidy supported shot price, operating years of the program, target population size for vaccination, shot location intensity, income and various public relations tools. The best model is selected by c-AIC, and varying and constant price elasticities are calculated from estimation results.

**Results:**

The vaccine uptake rate and the shot price have a negative relation. From the results of varying price elasticity, the demand for vaccination is elastic at municipalities with a shot price higher than 3,708 JPY (35.7 USD). Effects of public relations on the uptake rate are not found.

**Conclusions:**

It can be suggested that municipalities with a shot price higher than 3,708 JPY (35.7 USD) could subsidize more and reduce price to increase the demand for vaccination. Effects of public relations are not confirmed in this study, probably due to measurement errors of variables used for public relations, and studies at micro level exploring individual’s response to public relations would be required.

## Background

The administration of 23-valent pneumococcal polysaccharide vaccine (PPV) has been proven to be effective in reducing the incidence of invasive pneumococcal disease caused by Streptococcus pneumoniae (*S*. *pneumoniae*) among the elderly by 50% to 70% [1,2]. It is also effective in reducing mortality from severe community acquired pneumonia that requires hospitalization [3,4]. Several developed countries have implemented national pneumococcal vaccination programs for the elderly in order to prevent the disease and improve its outcomes [5-8], although the incidence of the disease varies worldwide [9].

Such programs target high uptake rates and coverages [10,11], for which subsidies and public relations (PR) are often organized in order to encourage the elderly to get vaccinated. However, the effects of subsidies and PR in publicly funded vaccination program have not been studied well in the literature. Al-Sukhni et al. (2008) found that in Canada, physician’s face to face advocacy inside the consultation room is important for the elderly to decide for influenza vaccination or pneumococcal vaccination [12]. Li, Norton, and Dow (2004) examined the threat-responsiveness hypothesis among the elderly and their decision to obtain influenza vaccination or pneumococcal vaccination in the U.S., and found that an increased associated mortality in the previous year does not significantly affect the demand for pneumococcal vaccination but significantly affect the demand for influenza vaccination [13]. Nevertheless, no studies have been reported on the effect of subsidy, or the price elasticity of demand for pneumococcal vaccination. This lack of knowledge is probably due to the fact that usually, immunization programs set fixed subsidized vaccine price for target population, and this makes it difficult to observe the consumer’s response to price changes.

In Japan, despite of the fact that pneumonia has been the fourth leading cause of death among the elderly aged 65 or over since 1975 [14], and that *S*. *pneumoniae* is the most common etiologic agent of community acquired pneumonia which accounts for 38.7% of such cases [15], a national pneumococcal vaccination program is yet to be set. In 2001, however, one town initiated a pneumococcal vaccination program for the elderly, under which aged inhabitants were encouraged to receive a subsidized PPV shot. Subsequently, several municipalities introduced similar programs, and by 2007, those amounted to 63 out of all 1,821 municipalities [16]. These programs set various levels of subsidized shot price and organized various PR at municipality’s own discretion, which enable us to observe consumers’ response, that is, the uptake rate, to subsidized price and PR.

We take advantage of this Japanese context, and aim to estimate a demand function for pneumococcal vaccination with price elasticity and effects of various PR tools on the uptake rate. The results of this study should deepen our understanding of consumer’s behavior towards preventive health care, and have implications for health managers in charge of vaccination programs to organize more effective programs, not only in Japan but also in other developed countries.

## Methods

A questionnaire survey was carried out to all municipal authorities operating pneumococcal vaccination programs in 2008. At the time of the survey, 63 municipalities operated programs and the response rate was 100%. In the survey, following questions were asked: operating years of the program, definition and size of the target population, uptake population, price to get one vaccine shot, number of health facilities providing shots, and PR tools taken for promoting vaccination. The type of PR tools included:

### To target population

“House-to-house delivery of brochures (PR1)”, “Distribution of brochures at health facilities (PR2)”, “Distribution of brochures at public facilities (PR3)”, “Insert articles in newsletters (PR4)”, “Upload information to websites (PR5)”, “Hold public events (PR6)”, “Local cable broadcasting (PR7)”

### To physicians

“Distribution of brochures to their health facilities (PR8)”, “Hold information events (PR9)”, “Communication in regular meetings (PR10)”

### To health nurses & care givers

“Distribution of brochures to their health facilities (PR11)”, “Hold information events (PR12)”, “Communication in regular meetings (PR13)”

These questions were asked on a Yes/No basis. With this municipal based data, we assume each municipality as a market for vaccination and examine the effects of PR tools implemented by the municipality and subsidy supported shot price on the vaccine uptake rate.

Descriptive statistics are reported in Table1. Due to missing or inadequacy values for data analysis, 39 out of the 63 municipalities were chosen for the estimation. Inadequacy values included the price of a shot if it was changed many times during the operating years of the program, or if the municipality had set fixed subsidies to health care providers and had them decide their own retail shot prices that reflected discretional technical service fees. The “Uptake rate” is the uptake population divided by the target population for vaccination during the operating years of the program. A municipality with 0.991 in the maximum shows that vaccination program had run successfully, meaning that 99.1% of the target population had been vaccinated. The “Shot price” is individual cost burden for receiving one shot. The 0 in the minimum means that the individual shot price is fully covered by the subsidy, and on the other hand, 6.500 in the maximum implies that the price is not fully covered and an individual has to pay 6,500 Japanese yen (JPY) (62.5 USD; 1USD = 104JPY in 2008 annual average) for vaccination. The “Operating years” is the length of operating the program. Note the maximum is 5 years, meaning that the data taken is the first 5 years from the beginning of the vaccine program, therefore there is no individual who has taken the shot twice. This is because effectiveness of PPV shot is said to last for 5 years [1] and revaccination was prohibited in Japan at the time of the survey. That is to say, the demand for vaccination would be decided by the shot price, PR, etc., and not by some sentiment from previous shot experience. The “Target population” is defined by the age criteria: ≥ 65, ≥ 70, or ≥ 75 years old, depending on the municipality. The difference of 0.217 in the minimum and 44.353 in the maximum is not because of age criteria chosen by municipalities, such as ≥ 75 in the minimum and ≥ 65 in the maximum, but of the population size: one with 0.217 shows a small village and the other with 44.353 shows a big city. The “Location intensity” is the number of health facilities providing vaccination per *km*^2^ in the municipality. The “Income” is the average income per capita by municipality obtained from System of Social and Demographic Statistics by Statistics Bureau [17]. The demand function requires a budget constraint information such as income [18], however, we cannot define the value from our municipal based questionnaire survey data. And there was no available income data on the targeted elderly but only of the entire citizen in the municipality. The “PR1 to 13” are binary or dummy variables which take 1 if the municipality implements the PR tool and take 0 otherwise. 79.5% of the municipalities take PR4, while only 7.7% take PR9 and PR12. “To target population”, “To physicians”, “To nurses & care givers” and “Total” show the number of PR tools implemented by the municipality during the operating years of the program; PR1 to PR7, PR8 to PR10, PR11 to PR13 and PR1 to PR13, respectively. 3.667 in the mean of “To target population” describes that municipalities implement on the average of 3 or 4 PR tools among PR1 to PR7.

**Table 1:** Descriptive statistics

					***n*** **= 39**
	**Ratio**	**Mean**	**Min.**	**Max.**	**S.d.**
Uptake rate		0.268	0.007	0.991	0.223
Shot price (1,000 JPY)		3.556	0.000	6.500	1.310
Operating years (year)		2.128	1.000	5.000	1.341
Target population (1,000 persons)		3.625	0.217	44.353	7.139
Location intensity (per km^2^)		0.474	0.002	9.966	1.599
Income (1,000,000 JPY)		1.233	0.564	4.984	0.732
Implemented PR tools:					
To target population (Count PR1-7)		3.667	1.000	7.000	1.284
PR1	0.564				
PR2	0.718				
PR3	0.538				
PR4	0.795				
PR5	0.513				
PR6	0.205				
PR7	0.333				
To physicians (Count PR8-10)		0.897	0.000	3.000	0.968
PR8	0.487				
PR9	0.077				
PR10	0.333				
To nurses & care givers (Count PR11-13)		0.385	0.000	2.000	0.747
PR11	0.179				
PR12	0.077				
PR13	0.128				
Total (Count PR1-13)		4.949	1.000	11.000	2.176

The “Uptake rate” is the response variable in our demand function, and we have “Shot price” and “Income” as explanatory variables [18]. As considered to affect the response variable, “Operating years”, “Target population”, “Location intensity” and “PR1 to 13”; “To target population”, “To physicians”, and “To nurses & care givers”, and “Total” are also included into the demand function. If the program has been operating for a long period of time, it would have become more common and high uptake rate is expected, in regards to “Operating years”. The “Target population” is regarded as nature of works which municipal authorities have to undertake to promote vaccination. It would be an extreme example, but making an effort to increase the uptake rate for a target population of one person is much easier than for a target population of 10,000 persons. The “Location intensity” is regarded as the non-cash price such as travel or time cost which has been proven to be significant in the demand for health care [19] including vaccination [20]. Investigating the effects of PR tools on the uptake rate is the aim of this study, therefore, we include “PR1 to 13” as dummy variables to examine which PR tools are effective to increase the uptake rate. In addition, the number of PR tools implemented by the municipality is considered as another PR scale, because it may reflect the intensity of PR within the municipality.

We assume a linier demand function, then the estimation form is expressed as [21]:

(1) $$Y_{i}=\beta_{0}+\beta_{1}x_{1i}+\beta_{2}x_{2i}+\ldots +\beta_{p}x_{\mathrm{pi}}+\varepsilon_{i}\;\left(i=1,\ldots ,n\right)\text{,}$$

where *Y* is the response variable “Uptake rate” in this study, $x_{j}\left(j=1,\ldots ,p\right)$ are explanatory variables, $\beta_{j}\left(j=0,\ldots ,p\right)$ are the constant and coefficients, *ε* is the error term, and *n* is the sample size. Three models are estimated: “Shot price”, “Operating years”, “Target population”, “Location intensity”, “Income” in all three models; “Total” is added in model 1; “To target population”, “To physicians” and “To nurses & care givers” are added in model 2; and “PR1 to 13” are added in model 3.

In order to examine the effects of explanatory variables, especially the effects of each PR tool on the “Uptake rate”, the best model is selected by c-AIC from model 3 as a full model, with a restriction to keep “Shot price” and “Income”. This is because “Shot price” and “Income” are conventional variables in the theory of demand [18]. Then c-AIC is calculated for all possible regressions with combinations of 16 explanatory variables. ‘c-AIC’ is the Akaike Information Criterion (AIC) for small sample data: the smaller the value of c-AIC, the better the model [22]. Sugiura (1987) suggested that AIC may perform poorly if there are too many parameters in relation to the size of the sample [23]. Our sample size is 39 and small, while parameters are 20, quite large, in model 3. The best model is created as model 4.

The price elasticity calculated from the estimation results of model 1, model 2, model 3, or model 4 is written as [18]:

(2) $$\delta_{v}=\frac{\partial Y}{\partial x_{1}}\cdot \frac{x_{1}}{Y}={\hat{\beta }}_{1}\cdot \frac{x_{1}}{\hat{Y}}$$

where *x*_1_ is the “Shot price”, ${\hat{\beta }}_{1}$ is the estimated coefficient of the “Shot price”, and $\hat{Y}$ is the expected value of the “Uptake rate” at *x*_1_. From equation (1), model 1, model 2, model 3, or model 4 is called as the varying elasticity model since the price elasticity can vary in response to changes in *x*_1_ and $\hat{Y}$[24]. Meanwhile, the constant elasticity model takes log(*x*_1_) and log(*Y*), instead of taking *x*_1_ and *Y* in the varying elasticity model [24]. That is:

(3) $$\log\left(Y_{i}\right)=\beta_{0}+\beta_{1}\log\left(x_{1i}\right)+\beta_{2}x_{\mathrm{pi}}+\ldots +\beta_{p}x_{\mathrm{pi}}+\varepsilon_{i}\;\left(i=1,\ldots ,n\right)$$

Then the constant price elasticity is expressed as:

(4) $$\delta_{c}=\frac{\partial Y}{\partial x_{1}}\cdot \frac{x_{1}}{Y}=\frac{\partial \log\left(Y\right)}{\partial \log\left(x_{1}\right)}={\hat{\beta }}_{1}$$

We focus to the best model, which is model 4, and estimate the varying and constant price elasticities, using a command “margins” in the software STATA12 [25]. The $\hat{Y}$ is calculated with all explanatory variables except *x*_1_, fixed at their means by using a command “atmeans”. Model 5 is created as the constant elasticity version of model 4 for reference.

Regarding research ethics, this study is not an experimental research nor carried on humans. It also falls outside of the guidelines of health research ethics in Japan. The data used for this study is openly available, and we received permission to use this data by all respondents’ municipalities.

## Results

Estimation results are listed in Table2. Model 1, model 2 and model 3 suggest that “Operating years”, “Target population”, “Location intensity”, “Income”, “Total”, “To target population”, “To physicians”, “To nurses & care givers” “PR1 to 13” do not affect the uptake rate, while “Operating years” in model 1 and “PR5” in model 3 are significant at 10%. “Shot price” is negatively significant in all four models, which confirms a negative relation between the “Shot price” and the “Uptake rate”.

**Table 2:** Estimation results

					***n*** **= 39**
	**Uptake rate**				**log (Uptake rate)**
	**Model 1**	**Model 2**	**Model 3**	**Model 4**	**Model 5**
Consent	0.557***	0.618***	0.641**	0.662***	−1.023***
Shot price (1,000 JPY)	−0.090***	−0.089***	−0.079**	−0.069***	
log (Shot price) (1,000 JPY)					−0.148*
Operating years (year)	0.042*	0.038	0.032		
Target population (1,000 persons)	−0.007	−0.003	0.000		
Location intensity (per km^2^)	−0.018	0.028	0.015		
Income (1,000,000 JPY)	0.075	−0.029	0.007	−0.016	−0.008
To target population (Count PR1-7)		−0.008			
PR1			0.058		
PR2			−0.117***	−0.117***	−0.762**
PR3			0.007		
PR4			−0.059		
PR5			−0.147*		
PR6			0.165		
PR7			0.101		
To physicaians (Count PR 8–10)		−0.056			
PR8			−0.036		
PR9			−0.027		
PR10			−0.145		
To nurses & care givers (Count PR11-13)		−0.006			
PR11			−0.167		
PR12			0.041		
PR13			0.295		
Total (Count PR1-13)	−0.024				
c-AIC	−3.608	2.611	49.812	−11.124	113.527
AIC	−8.408	−5.246	3.145	−12.942	111.709
Adjust R^2^	0.235	0.201	0.111	0.274	0.142

Signif codes: 0.01 ‘***’ 0.05’**’ 0.1’*’.

Comparing c-AIC, model 4 has the smallest value and is regarded as the best among four models. The AIC and adjusted R^2^ also imply that model 4 is the best, however, the difference in AIC between −8.408 in model 1 and −12.942 in model 4, 4.534, is smaller than the corresponding difference in c-AIC, 7.516, which tells us that c-AIC performs better than AIC in selecting the best model. The coefficient of the “Shot price” in model 4, −0.069, explains that the uptake rate is decreased by 6.9% with an increase of the shot price at 1,000 JPY (9.6 USD). Contrary to our expectations, “Operating years”, “Target population”, and “Location intensity” are not selected by c-AIC, and “Income” is not found statistically significant, which implies that these variables have little effect on the uptake rate. Only “PR2” is selected by c-AIC but its coefficient sign is negative, however. It is not clear whether any of these PR tools promotes the increase in uptake rate.

Model 5 is the estimated result of constant elasticity model. The coefficient of the “log(Shot price)”, −0.148, is regarded as the constant price elasticity, and it is larger than −1 and smaller than 0, which implies that the demand for vaccination is inelastic.

**Figure 1:**
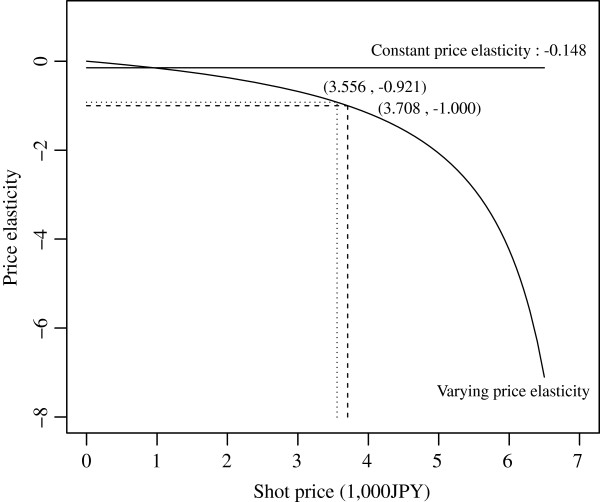
**Price elasticity Vs Shot price.** Relations between varying or constant price elasticity and shot price, according to the estimation results from model 4 or model 5, respectively. Solid lines show constant and varying price elasticities. Dashed and dotted lines show the unit elasticity and the elasticity at mean shot price on the varying price elasticity, respectively.

Figure1 describes varying and constant price elasticities calculated from estimation results of model 4 and model 5, respectively. From (1) and the negative coefficient value of the “Shot price” in model 4, −0.069, the varying price elasticity becomes negatively larger as “Shot price” takes larger positive value. The shot price is 3,708 JPY (35.7 USD) at unit elasticity, i.e., *δ*_*v*_ = −1. Therefore, the demand for vaccination is inelastic at the mean price of 3,556 JPY (34.2 USD), and becomes elastic at more than 3,708 JPY (35.7 USD).

## Discussion

We estimate the demand function for pneumococcal vaccination under subsidy programs incorporating the shot price, various PR tools and other factors considered to affect the demand, i.e., the uptake rate. The uptake rate is found to depend significantly on the shot price. Therefore, reducing the shot price by subsidy is an effective implementation to achieve higher coverage. Additionally, we estimated price elasticity of the demand, which has not been studied well in the literature. According to the varying price elasticity of demand, the demand is inelastic, more than – 1, when the shot price is reduced, supported by larger subsidy. And it is elastic, less than – 1, when the price is higher with smaller subsidy. Unitary elasticity is estimated at 3,708 JPY (35.7 USD), so municipalities offering higher than 3,708 JPY (35.7 USD) for a shot can expect substantial gains in uptake rates by reducing shot prices.

Since only subsidized shot price is available for our analysis, it is not possible to discuss the direct link between subsidy and demand. We can, however, give a probable breakdown of subsidy and shot price. The National Health Insurance price list gave 4,835 JPY (46.9 USD) for 23-valent PPV at the time of the survey, although municipalities or vaccination providers might have purchased at a discounted price for such public health program that is not covered by the National Health Insurance reimbursement. And arguably, it can be assumed that the technical service fee for administrating one shot levied by physicians is around 5,000 JPY (48.1 USD). Therefore, the municipality are likely to expend 10,000 JPY (96.2 USD) to 3,500 JPY (33.7 USD) per shot as subsidy in order to set the price of a shot at 0 JPY (0 USD) to 6,500 JPY (62.5 USD).

Although we anticipated a result that the demand increases by implementing more PR tools, their effectiveness is not found. Furthermore, any positive effect of each PR tool on the demand is not observed. However, we do not think that these results suggest that PR is ineffective in organizing vaccination programs. On the contrary, these failures could be attributable to the measurement errors of variables we used, which are difficulties inherent in this study. 13 PR tools had been asked on only a Yes/No basis in the survey because there was no a priori knowledge about PR practices in this context. One possible account is a lack of appraisal of actual contents of each PR tool. It can be assumed that the contents are divided into two types: information about arrangement and procedure of the program including the shot price; and information about risk and benefit of vaccination, that is, health education. PR tools containing price information could cause negative effects on the uptake rate. And PR tools containing health education information might also not work well as it may cause irrational response among people with aversion to vaccination. It is known that there is a negative attitude towards vaccination among Japanese health professionals compared to those of overseas [26]. Their aversion to vaccination has been firmly rooted by the anti vaccination campaign in the 1990s in Japan [27], where both the public [28] and physicians [26] have become to fear its adverse effects. Therefore, the use of PR in order to dispel this negative attitude could prompt the demand.

The effects of the operating years of the program and target population size to the uptake rate were not found, which could be explained that communicating to the aged inhabitants may be similar among the municipalities irrespective of time span or size. The location intensity may be failing in measuring the travel cost. The travel cost to the elderly may not be just direct distance to health facilities. It depends more on the access assured by public transportation, or consulting their home doctor to make their decision in their regular doctor visits, which cannot be caught by number of health facilities providing a shot per *km*^2^.

In addition to the lack of contents and information regarding PR tools and the travel cost, the number of usable observations of only 39 municipalities is small, even though we used the data set with 100% response rate and took statistical technique for small samples, i.e., c-AIC. This may be another reason why anticipated results were not found in this study.

It is notable that the estimated price elastic demand for pneumococcal vaccination contrasts with the price inelastic demand for influenza vaccination among similar population [29]. These two vaccinations for the elderly are also found differently in the threat-responsiveness hypothesis about demand in the U.S. [13]. Comparative study between the two vaccinations is awaited to deepen our understanding about this difference.

This study leaves a room for further study. Particularly, effects of PR on the demand would be of interest to academics as well as to health managers currently in practice. Our experience in this study suggests that rigorous PR measurements for estimating the demand function across diverse municipal programs is quite difficult unless any contextual opportunity is offered. Studies at micro level exploring individual’s response to PR could be one of the possible approaches to obtain some evidence.

## Conclusions

The elderly’s demand for pneumococcal vaccination under subsidy programs in Japan is found price-sensitive. Subsidy works, and setting the appropriate level of price for a shot is important in organizing such programs. High gains in uptake rates and coverage are expected by increasing subsidy when the price of a shot is higher than 3,708 JPY (35.7 USD). The role of PR or its effectiveness in obtaining high uptake rates and coverage could not be confirmed in this study.

## Competing interests

Authors declare there is no conflict of interest.

## Authors' contributions

MK, SH, and IO were involved in conception and design of this project, while MK and MY were involved with the implementation of the project and analysis and interpretation of the data. MK and MY have drafted the manuscripts while IO had provided critical comments. “All authors read and approved the final manuscript.”

## Pre-publication history

The pre-publication history for this paper can be accessed here:

http://www.biomedcentral.com/1472-6963/12/313/prepub
